# Analysis of outcomes of nab-paclitaxel compared to other taxanes in early breast cancer: A study revealing the critical role of calendar-time bias in real-world drug comparisons

**DOI:** 10.1016/j.breast.2026.104934

**Published:** 2026-09-25

**Authors:** Jingjing Xiao, Meiling Huang, Changjiao Yan, Zhe Wang, Ting Wang

**Affiliations:** Department of Thyroid, Breast and Vascular Surgery, Xijing Hospital, The Fourth Military Medical University, Xi'an, Shaanxi, China

**Keywords:** Breast cancer, Nab-paclitaxel, Real-world study, Calendar-time bias, Propensity score matching

## Abstract

**Objective:**

To compare long-term survival outcomes of nab-paclitaxel versus other taxanes in early breast cancer and to evaluate the impact of calendar-time bias in real-world studies.

**Methods:**

We enrolled 9217 early invasive breast cancer patients (2007–2024) divided into nab-paclitaxel (n = 3860) and other taxanes (n = 5357) groups. The primary analysis employed three separate Cox models: Model 1 (unadjusted for time), Model 2 (adjusted for year of diagnosis), and Model 3 (adjusted for COVID-19 periods). Sensitivity analyses included propensity score matching, overlap-period restriction, restricted cubic splines, calendar-period stratification, and multiple imputation. Endpoints were overall survival (OS), disease-free survival (DFS), and distant metastasis-free survival (DMFS).

**Results:**

Median diagnosis year was 2021 for nab-paclitaxel vs. 2015 for other taxanes (P < 0.001). Without time adjustment, other taxanes showed higher recurrence and metastasis risk (DFS: HR = 1.366, P < 0.001; DMFS: HR = 1.294, P = 0.004). After adjusting for year categories, the effect disappeared (DFS: HR = 1.068, P = 0.536; DMFS: HR = 0.970, P = 0.789; OS: HR = 1.015, P = 0.932). After adjusting for COVID-19 periods, DFS showed HR = 1.259 (P = 0.018), DMFS showed HR = 1.174 (P = 0.125), and OS showed HR = 1.148 (P = 0.378). Year of diagnosis was a strong independent prognostic factor. All sensitivity analyses for survival endpoints consistently confirmed the absence of a significant treatment effect.

**Conclusion:**

After adjusting for calendar-time bias using categorical year adjustment, no significant differences in long-term survival were observed between nab-paclitaxel and other taxanes in patients with early breast cancer. This study highlights the critical role of calendar-time bias in long-term real-world comparative studies, and provides important methodological guidance for future similar research.

## Highlights

• Calendar-time bias can produce spurious treatment effects in long-term real-world studies.
• After adjusting for year of diagnosis as a categorical variable, no significant differences in long-term survival were observed between nab-paclitaxel and other taxanes in early breast cancer.
• Year of diagnosis was a strong independent prognostic factor.
• Calendar-time bias should be adjusted for in long-term real-world comparative research.

## Graphical abstract

**Figure:**
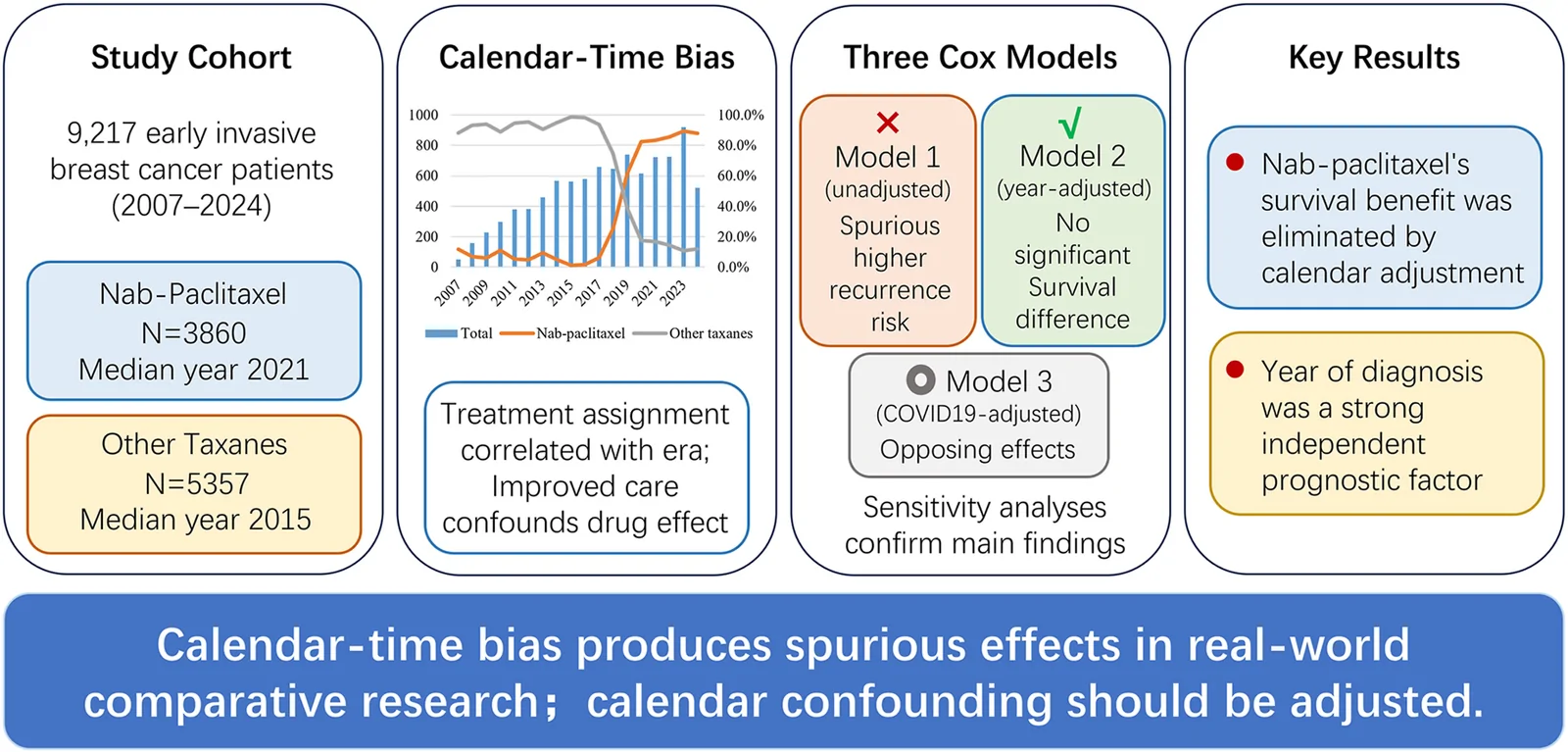

## 1 Introduction

Breast cancer is the most common malignancy among women worldwide, with an incidence that continues to rise, posing a serious threat to women's health [1]. For patients with early breast cancer, a comprehensive treatment strategy centered on surgery, combined with chemotherapy, radiotherapy, endocrine therapy, and targeted therapy, is key to improving prognosis [2]. Chemotherapy plays an important role in reducing the risk of recurrence and death, and taxane-based regimens have become a standard treatment option for early breast cancer, particularly in high-risk patients [3].

Conventional taxanes, such as paclitaxel and docetaxel, inhibit mitotic cell division by stabilizing microtubules and have well-established efficacy [4]. However, their poor water solubility necessitates the use of solubilizing agents, which can cause severe allergic reactions and require premedication with antihistamines and corticosteroids. Nab-paclitaxel (albumin-bound paclitaxel) uses human serum albumin as a carrier, eliminating the need for chemical solvents, and is expected to achieve higher intratumoral drug concentrations and better therapeutic effects [5]. Several clinical studies have suggested that, in the neoadjuvant setting, nab-paclitaxel significantly improves the pathological complete response rate compared with solvent-based paclitaxel [6]. However, most available evidence comes from neoadjuvant or metastatic breast cancer trials, predominantly randomized controlled trials [7]. In the broader real-world setting of neoadjuvant and adjuvant therapy for early breast cancer, data on long-term survival outcomes—particularly disease-free survival and overall survival—comparing different taxanes remain limited and show heterogeneous results [8,9].

Real-world studies can complement clinical trials by providing information on populations not covered and reflecting actual clinical practice, offering unique value. Notably, when real-world studies span long periods, a critical but easily overlooked confounder—calendar-time bias or period effect bias—can seriously compromise the reliability of conclusions. Calendar-time bias refers to the confounding effect arising from the association between treatment assignment and calendar period, where improvements in diagnosis, treatment, and supportive care over time may drive observed outcome differences. Over time, breast cancer diagnosis and treatment strategies, supportive care, and access to subsequent therapies have evolved, and these changes are associated with treatment selection [10]. Without adjustment for temporal factors, observed differences in outcomes may reflect improvements in medical care over time rather than differences in drug efficacy.

This study aims to systematically compare the long-term outcomes of nab-paclitaxel versus other taxanes in patients with early invasive breast cancer using large-sample, single-center real-world data, and to examine the impact of calendar-time bias using multiple analytical approaches. The findings are intended to serve as a methodological example for long-term real-world study designs and to highlight the impact of calendar-time bias on comparative effectiveness research.

## 2 Materials and methods

### 2.1 Study population

This study collected clinicopathological data from patients diagnosed with breast cancer and treated at Xijing Hospital between January 2007 and July 2024. Patients were selected according to the following inclusion and exclusion criteria. Inclusion criteria: (1) histopathologically confirmed invasive breast cancer; (2) female; (3) age at diagnosis ≥18 years; (4) received chemotherapy at initial treatment with a taxane-containing regimen; (5) underwent surgical treatment; (6) no distant metastasis on systemic evaluation. Exclusion criteria: (1) male; (2) bilateral breast cancer; (3) inflammatory breast cancer; (4) stage IV at initial diagnosis; (5) primary breast tumor not operable; (6) missing clinical data; (7) less than 4 cycles of initial taxane therapy. After applying the inclusion and exclusion criteria, 9217 patients were finally enrolled. The data screening flow diagram is shown in Supplementary Fig. S1.

### 2.2 Data collection and definitions

Collected clinical data included: date of diagnosis, age at diagnosis, menstrual status, primary tumor site, clinical TNM stage, pathological type, pathological WHO grade, ER and PR immunohistochemistry results, HER2 immunohistochemistry results, HER2 FISH results, Ki-67, immunohistochemical subtype, surgical procedure, neoadjuvant chemotherapy, endocrine therapy, radiotherapy, targeted therapy, type of initial taxane, and follow-up data. Date of diagnosis was defined as the date of pathological confirmation. Hormone receptor (HR) positivity was defined as ER/PR expression >1%. HER2 positivity was defined as immunohistochemistry 3+ or positive fluorescence in situ hybridization (FISH) result. Clinical staging used the AJCC 8th edition breast cancer staging system. Immunohistochemical subtyping was based on the St. Gallen consensus, with an ER/PR threshold of 1% and a Ki-67 threshold of 14%, which was consistently applied throughout the study period to maintain uniform classification across different calendar years.

All patients were divided into the nab-paclitaxel group and the other taxanes group. The nab-paclitaxel group included patients whose initial chemotherapy regimen contained nab-paclitaxel. The other taxanes group included patients whose chemotherapy regimen contained docetaxel or other types of paclitaxel but not nab-paclitaxel. The primary endpoint was overall survival (OS). Secondary endpoints were disease-free survival (DFS) and distant metastasis-free survival (DMFS). OS was defined as the time from pathological diagnosis to death from any cause or last follow-up. DFS was defined as the time from pathological diagnosis to first disease recurrence (local recurrence or distant metastasis). DMFS was defined as the time from pathological diagnosis to first distant metastasis.

This study was a retrospective study. Data collection followed the tenets of the Declaration of Helsinki and was approved by the Ethics Committee of Xijing Hospital (Ethics Approval Number: No. KY20232266-F-1).

### 2.3 Statistical methods

All statistical analyses were performed using Stata 19.0 (StataCorp, College Station, TX, USA). For comparisons between groups, categorical variables were analyzed using the chi-square test or Fisher's exact test. Continuous variables following a normal distribution were compared using the independent samples *t*-test, while those not following a normal distribution were compared using the Mann-Whitney *U* test. For categorical variables with “unknown” or missing data, these were set as a separate category and included in all statistical analyses to maximize sample size and allow assessment of whether missing data patterns were associated with non-random prognosis. Covariates for multivariable adjustment were selected based on clinical relevance and established prognostic factors in breast cancer, including age, tumor stage, nodal status, tumor grade, proliferation index, molecular subtype, and treatment-related factors. Interaction terms were included in Cox regression models to test for interactions and explore the consistency of treatment-outcome associations across different clinical characteristics. All tests were two-sided, and a P-value <0.05 was considered statistically significant.

### 2.4 Survival analysis and calendar-time bias adjustment strategy

Kaplan-Meier methods were used for survival analysis, and between-group comparisons were performed using the log-rank test. To evaluate the impact of calendar-time confounding factors, we constructed three separate multivariate Cox proportional hazards models.

Model 1 (unadjusted for time) included all clinicopathological and treatment covariates selected based on clinical relevance (age, cT stage, cN stage, WHO grade, Ki-67, immunohistochemical subtype, breast surgery type, axillary surgery type, neoadjuvant chemotherapy, endocrine therapy, targeted therapy, radiotherapy) but did not include any time variables.

Model 2 (adjusted for year) added year of diagnosis as a categorical variable with four periods (2007–2012, 2013–2017, 2018–2019, and 2020–2024) to Model 1 to adjust for confounding effects from long-term medical advances. The categorization was chosen based on the evolution of clinical guidelines and treatment paradigms in China, ensuring sufficient sample size and events in each period while capturing clinically meaningful shifts in treatment approaches over time.

Model 3 (adjusted for COVID-19 periods) replaced the year categorical variable with COVID-19 periods (pre-pandemic, before January 2020; pandemic, January 2020 to March 2023; post-pandemic period, after April 2023) to capture the potential impact of the pandemic-related healthcare disruptions. Due to strong collinearity, year categories and COVID-19 periods could not be included simultaneously in the same model.

### 2.5 Propensity score matching and sensitivity analyses

To further control for confounding and to validate the robustness of the primary findings, 1:1 propensity score matching was performed. Matching variables included age, menstrual status, clinical diagnosis, cT stage, cN stage, WHO grade, hormone receptor status, HER2 status, Ki-67, breast surgery type, axillary surgery type, neoadjuvant chemotherapy, endocrine therapy, targeted therapy, radiotherapy, year of diagnosis, and COVID-19 period. The caliper value was set at 0.02, and random matching was enabled to minimize bias. After matching, standardized mean differences (SMD) were used to assess covariate balance, with SMD <0.1 indicating good balance. In the matched cohort, stratified Cox regression was performed to estimate the treatment effect.

Additional sensitivity analyses were performed to assess the robustness of our findings. (1) Overlap-period restriction (2018–2019): Analysis restricted to patients diagnosed in the period when both taxane strategies were used with sufficient overlap. (2) Year of diagnosis as restricted cubic splines (RCS): To allow flexible, non-linear adjustment for calendar year. (3) Calendar-period stratified analysis: Stratified by calendar period to control for period effects. (4) Overlap-period PSM: PSM restricted to the 2018–2019 overlap period. (5) Multiple imputation (MI): For missing covariate data using chained equations with 20 imputed datasets. Variables with moderate missing proportions (5%–30%) were imputed, including WHO grade (24.5%), Ki-67 (9.2%), HER2 (6.7%), endocrine therapy (29.4%), and targeted therapy (13.8%). Variables with very low missing proportions (<5%, including T stage, N stage, hormone receptor status) and derived variables (immunohistochemical subtype) were not imputed; radiotherapy was not imputed due to its high missing proportion (54.8%).

## 3 Results

### 3.1 Baseline characteristics of the overall population

A total of 9217 patients were included in the analysis. The median age of the overall population was 48 years (IQR 41–55). Premenopausal patients accounted for 61.3% of the cohort. Clinical stage II was the most common (66.6%). Breast-conserving surgery was performed in 22.1% of patients, total mastectomy in 68.0%, and mastectomy with reconstruction in 10.0%. Neoadjuvant chemotherapy was administered to 49.6% of patients, endocrine therapy to 40.7%, targeted therapy to 23.7%, and radiotherapy to 45.2%. The median follow-up time for all patients was 50 months (IQR 24–93 months). Median follow-up was 25 months (IQR 17–42) for the nab-paclitaxel group and 87 months (IQR 54–116) for the other taxanes group. Event numbers and survival rates at fixed time points by treatment group are presented in Supplementary Table S1. The baseline characteristics of the overall population are detailed in Table 1.

**Table 1:** Baseline characteristics of the overall population and treatment groups.

		Total		Nab-paclitaxel		Other taxanes		P
		N(9217)	%	N(3860)	%	N(5357)	%	
Year of diagnosis (quartiles)		2018(2014-2021)		2021(2020-2023)		2015(2012-2017)		<0.001
Year of diagnosis	2007-2012	1500	16.3%	102a	2.6%	1398b	26.1%	<0.001
	2013-2017	2824	30.6%	130a	3.4%	2694b	50.3%	
	2018-2019	1387	15.0%	618a	16.0%	769b	14.4%	
	2020-2024	3506	38.0%	3010a	78.0%	496b	9.3%	
COVID-19	Pre-pandemic	5423	58.8%	697a	12.9%	4726b	87.1%	<0.001
	Pandemic	2584	28.0%	2081a	80.5%	503b	19.5%	
	Post-pandemic periods	1210	13.1%	1082a	89.4%	128b	10.6%	
Age (quartiles)		48(41-55)		48(40-55)		48(42-55)		0.958
Age	≤35	1050	11.4%	518a	13.4%	532b	9.9%	<0.001
	36-45	2677	29.0%	1043a	27.0%	1634b	30.5%	
	46-55	3312	35.9%	1350a	35.0%	1962a	36.6%	
	56-69	2056	22.3%	886a	23.0%	1170a	21.8%	
	≥70	122	1.3%	63a	1.6%	59b	1.1%	
Menstrual status	Premenopausal	5647	61.3%	2337a	60.5%	3310a	61.8%	0.226
	Postmenopausal	3570	38.7%	1523a	39.5%	2047a	38.2%	
Clinical diagnosis	Left-sided breast cancer	4809	52.2%	1988a	51.5%	2821a	52.7%	0.272
	Right-sided breast cancer	4408	47.8%	1872a	48.5%	2536a	47.3%	
cT	0	27	0.3%	16a	0.4%	11a	0.2%	<0.001
	1	2877	31.2%	1144a	29.6%	1733b	32.4%	
	2	5063	54.9%	2101a	54.4%	2962a	55.3%	
	3	719	7.8%	380a	9.8%	339b	6.3%	
	4	309	3.4%	152a	3.9%	157b	2.9%	
	Unknown	222	2.4%	67a	1.7%	155b	2.9%	
cN	0	4342	47.1%	1882a	48.8%	2460b	45.9%	<0.001
	1	4288	46.5%	1742a	45.1%	2546b	47.5%	
	2	236	2.6%	44a	1.1%	192b	3.6%	
	3	323	3.5%	183a	4.7%	140b	2.6%	
	Unknown	28	0.3%	9a	0.2%	19a	0.4%	
Clinical stage	1	1672	18.1%	676a	17.5%	996a	18.6%	<0.001
	2	6142	66.6%	2579a	66.8%	3563a	66.5%	
	3	1177	12.8%	538a	13.9%	639b	11.9%	
	Unknown	226	2.5%	67a	1.7%	159b	3.0%	
WHO grade	1	438	4.8%	197a	5.1%	241a	4.5%	<0.001
	2	4707	51.1%	2181a	56.5%	2526b	47.2%	
	3	1814	19.7%	845a	21.9%	969b	18.1%	
	Unknown	2258	24.5%	637a	16.5%	1621b	30.3%	
Hormone receptor (ER/PR)	Positive	6346	68.9%	2659a	68.9%	3687a	68.8%	<0.001
	Negative	2753	29.9%	1185a	30.7%	1568a	29.3%	
	Unknown	118	1.3%	16a	0.4%	102b	1.9%	
HER-2	Positive	2832	30.7%	1296a	33.6%	1536b	28.7%	<0.001
	Negative	5763	62.5%	2419a	62.7%	3344a	62.4%	
	Unknown	622	6.7%	145a	3.8%	477b	8.9%	
Ki-67	≤14%	1305	14.2%	494a	12.8%	811b	15.1%	<0.001
	15%-50%	5138	55.7%	2258a	58.5%	2880b	53.8%	
	>50%	1929	20.9%	1040a	26.9%	889b	16.6%	
	Unknown	845	9.2%	68a	1.8%	777b	14.5%	
Immunohistochemical subtyping	Luminal A	971	10.5%	367a	9.5%	604b	11.3%	<0.001
	Luminal B	3021	32.8%	1380a	35.8%	1641b	30.6%	
	HER2+(HR-)	1190	12.9%	495a	12.8%	695a	13.0%	
	HER2+(HR+)	1635	17.7%	798a	20.7%	837b	15.6%	
	Triple-negative	1466	15.9%	659a	17.1%	807b	15.1%	
	Unknown	934	10.1%	161a	4.2%	773b	14.4%	
Breast surgery type	Breast-conserving surgery	2036	22.1%	890a	23.1%	1146a	21.4%	<0.001
	Mastectomy	6263	68.0%	2385a	61.8%	3878b	72.4%	
	Mastectomy with reconstruction	918	10.0%	585a	15.2%	333b	6.2%	
Axillary surgery type	Sentinel lymph node biopsy	2624	28.5%	1519a	39.4%	1105b	20.6%	<0.001
	Axillary lymph node dissection	6593	71.5%	2341a	60.6%	4252b	79.4%	
Neoadjuvant chemotherapy	Yes	4569	49.6%	2269a	58.8%	2300b	42.9%	<0.001
	No	4648	50.4%	1591a	41.2%	3057b	57.1%	
Endocrine therapy	Yes	3751	40.7%	1733a	44.9%	2018b	37.7%	<0.001
	No	2753	29.9%	1185a	30.7%	1568a	29.3%	
	Unknown	2713	29.4%	942a	24.4%	1771b	33.1%	
HER2-targeted therapy	Yes	2181	23.7%	1213a	31.4%	968b	18.1%	<0.001
	No	5763	62.5%	2419a	62.7%	3344a	62.4%	
	Unknown	1273	13.8%	228a	5.9%	1045b	19.5%	
Radiotherapy	Yes	4168	45.2%	1485a	38.5%	2683b	50.1%	<0.001
	Unknown	5049	54.8%	2375a	61.5%	2674b	49.9%	

### 3.2 Baseline comparison and temporal distribution between the two groups

Patients were divided into the nab-paclitaxel group (n = 3860) and the other taxanes group (n = 5357) according to the initial taxane regimen. In the other taxanes group, 5296 patients (98.9%) received docetaxel and 61 patients (1.1%) received solvent-based paclitaxel. Differences in multiple baseline characteristics were observed between the two groups (Table 1).

The median year of diagnosis was 2021 (IQR 2020–2023) for the nab-paclitaxel group and 2015 (IQR 2012–2017) for the other taxanes group (P < 0.001). As shown in Fig. 1 and Supplementary Table S2, between 2007 and 2017, other taxanes dominated (accounting for >88% of regimens). From 2018 onward, the use of nab paclitaxel increased rapidly and became the predominant drug after 2020 (accounting for >82% of regimens). Categorized into four periods, 78.0% of nab-paclitaxel patients were diagnosed in 2020–2024, whereas 76.4% of other taxanes patients were diagnosed in 2007–2017 (P < 0.001). Similarly, the proportion of patients in the pandemic and post-pandemic periods was higher in the nab-paclitaxel group than in the other taxanes group (P < 0.001).

**Fig. 1:**
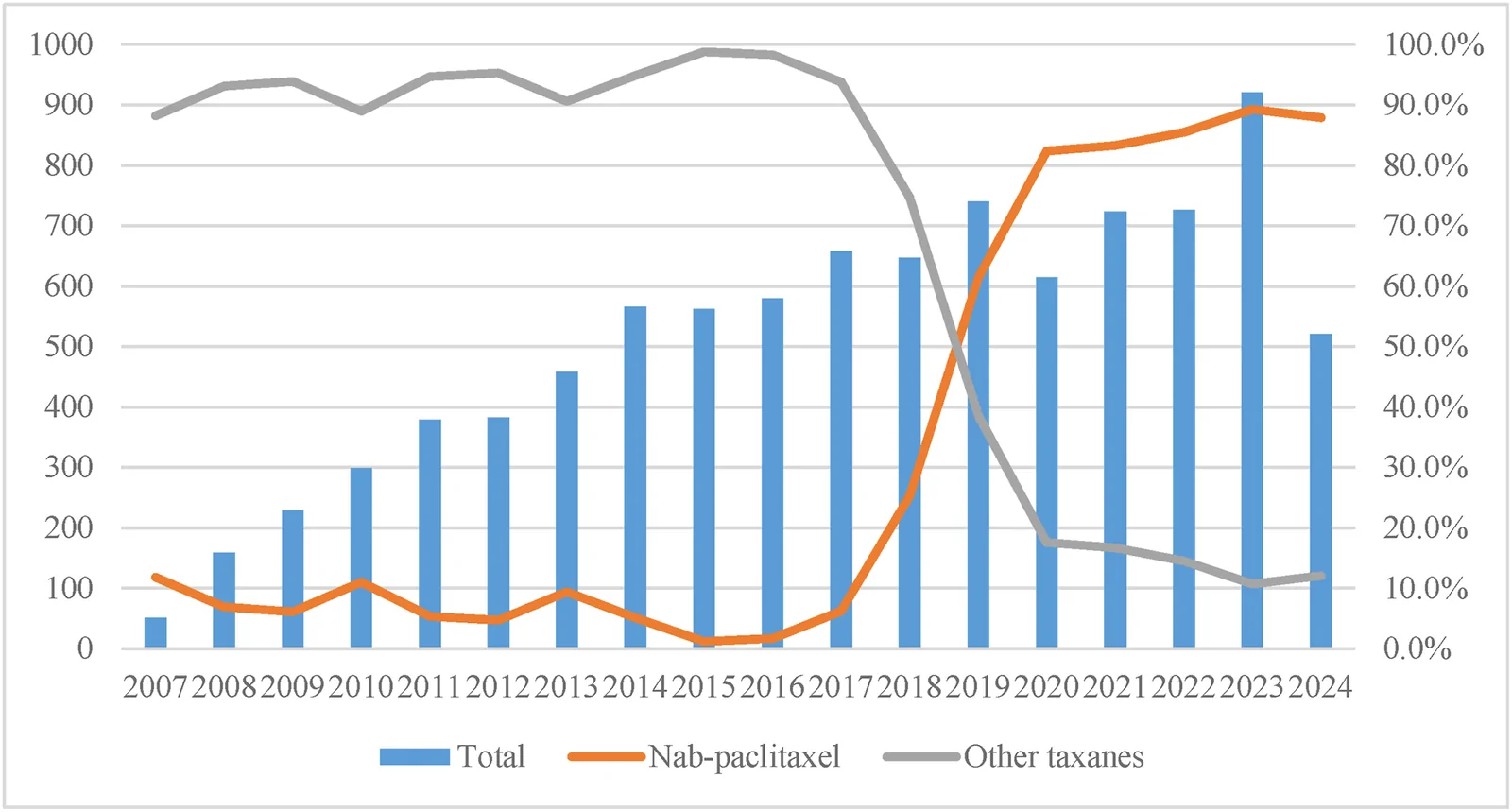
Temporal trends in the usage of study drugs.

### 3.3 Multivariable cox regression

To evaluate the impact of calendar-time confounding, we constructed three separate multivariable Cox models (Table 2).

**Table 2:** Multivariable Cox regression results from three separate models: unadjusted (Model 1), adjusted for year of diagnosis as 4-category variable (Model 2), and adjusted for COVID-19 periods (Model 3).

			Unadjusted for year and COVID-19		Adjusted for year		Adjusted for COVID-19	
			P	HR (95.0% CI)	P	HR (95.0% CI)	P	HR (95.0% CI)
COX-DFS	Taxane type	Nab-paclitaxel		1		1		1
		Other taxanes	<0.001	1.366(1.16-1.608)	0.536	1.068(0.867-1.316)	0.018	1.259(1.04-1.524)
	Year of diagnosis	2007-2012	—		<0.001		—	
		2013-2017	—	—	0.001	0.768(0.655-0.901)	—	—
		2018-2019	—	—	<0.001	0.503(0.392-0.645)	—	—
		2020-2024	—	—	<0.001	0.528(0.392-0.71)	—	—
	COVID-19	Pre-pandemic	—		—		0.088	
		Pandemic	—	—	—	—	0.395	0.897(0.699-1.152)
		Post-pandemic period	—	—	—	—	0.03	0.556(0.328-0.944)
COX-DMFS	Taxane type	Nab-paclitaxel		1		1		1
		Other taxanes	0.004	1.294(1.084-1.544)	0.789	0.97(0.775-1.214)	0.125	1.174(0.956-1.441)
	Year of diagnosis	2007-2012	—		<0.001		—	
		2013-2017	—	—	0.002	0.764(0.644-0.905)	—	—
		2018-2019	—	—	<0.001	0.471(0.359-0.617)	—	—
		2020-2024	—	—	<0.001	0.487(0.352-0.673)	—	—
	COVID-19	Pre-pandemic	—		—		0.071	
		Pandemic	—	—	—	—	0.294	0.863(0.655-1.136)
		Post-pandemic period	—	—	—	—	0.027	0.491(0.261-0.92)
COX-OS	Taxane type	Nab-paclitaxel		1		1		1
		Other taxanes	0.532	1.087(0.836-1.414)	0.932	1.015(0.729-1.412)	0.378	1.148(0.844-1.563)
	Year of diagnosis	2007-2012	—		<0.001		—	
		2013-2017	—	—	<0.001	0.52(0.409-0.661)	—	—
		2018-2019	—	—	<0.001	0.421(0.287-0.617)	—	—
		2020-2024	—	—	0.028	0.583(0.361-0.943)	—	—
	COVID-19	Pre-pandemic	—		—		0.76	
		Pandemic	—	—	—	—	0.556	1.137(0.741-1.747)
		Post-pandemic period	—	—	—	—	0.568	1.291(0.537-3.102)

Model 1 (unadjusted for time): Without including any time variables, other taxanes were associated with a higher risk of recurrence/metastasis compared with nab-paclitaxel: HR for DFS = 1.366 (95% CI 1.160–1.608, P < 0.001); HR for DMFS = 1.294 (95% CI 1.084–1.544, P = 0.004). No significant difference was observed for OS (HR = 1.087, 95% CI 0.836–1.414, P = 0.532).

Model 2 (adjusted for year as categorical variable): After including year of diagnosis as a categorical variable with four periods, the treatment effect was no longer statistically significant for all endpoints. The HR for DFS was 1.068 (95% CI 0.867–1.316, P = 0.536), the HR for DMFS was 0.970 (95% CI 0.775–1.214, P = 0.789), and the HR for OS was 1.015 (95% CI 0.729–1.412, P = 0.932). Year of diagnosis was a strong independent prognostic factor: compared with the 2007–2012 period, the 2013–2017 period was associated with HRs of 0.768 (P = 0.001) for DFS, 0.764 (P = 0.002) for DMFS, and 0.520 (P < 0.001) for OS; the 2018–2019 period was associated with HRs of 0.503 (P < 0.001) for DFS, 0.471 (P < 0.001) for DMFS, and 0.421 (P < 0.001) for OS; the 2020–2024 period was associated with HRs of 0.528 (P < 0.001) for DFS, 0.487 (P < 0.001) for DMFS, and 0.583 (P = 0.028) for OS.

Model 3 (adjusted for COVID-19 periods): After adjusting for COVID-19 periods, DFS, DMFS, and OS showed HRs of 1.259 (95% CI 1.040–1.524, P = 0.018), 1.174 (95% CI 0.956–1.441, P = 0.125), and 1.148 (95% CI 0.844–1.563, P = 0.378), respectively. The pandemic period was not significantly associated with prognosis compared with the pre-pandemic period for any endpoint (DFS: P = 0.395; DMFS: P = 0.294; OS: P = 0.556). The post-pandemic period was associated with better DFS (HR = 0.556, P = 0.030) and DMFS (HR = 0.491, P = 0.027), but not with OS (P = 0.568). The full models including all covariates are presented in Supplementary Table S3–S5.

### 3.4 Survival curves stratified by COVID-19 periods

Kaplan-Meier survival curves were plotted stratified by COVID-19 period (Fig. 2). Patients diagnosed during the post-pandemic recovery period had the highest survival estimates throughout follow-up. Patients diagnosed during the pandemic had higher survival estimates early during follow-up than those diagnosed pre-pandemic. However, the pandemic-period curve crossed below the pre-pandemic curve at a later time point during follow-up and remained lower thereafter. Overall differences among the three periods were statistically significant for DFS (P < 0.001) and DMFS (P < 0.001), but not for OS (P = 0.215).

**Fig. 2:**
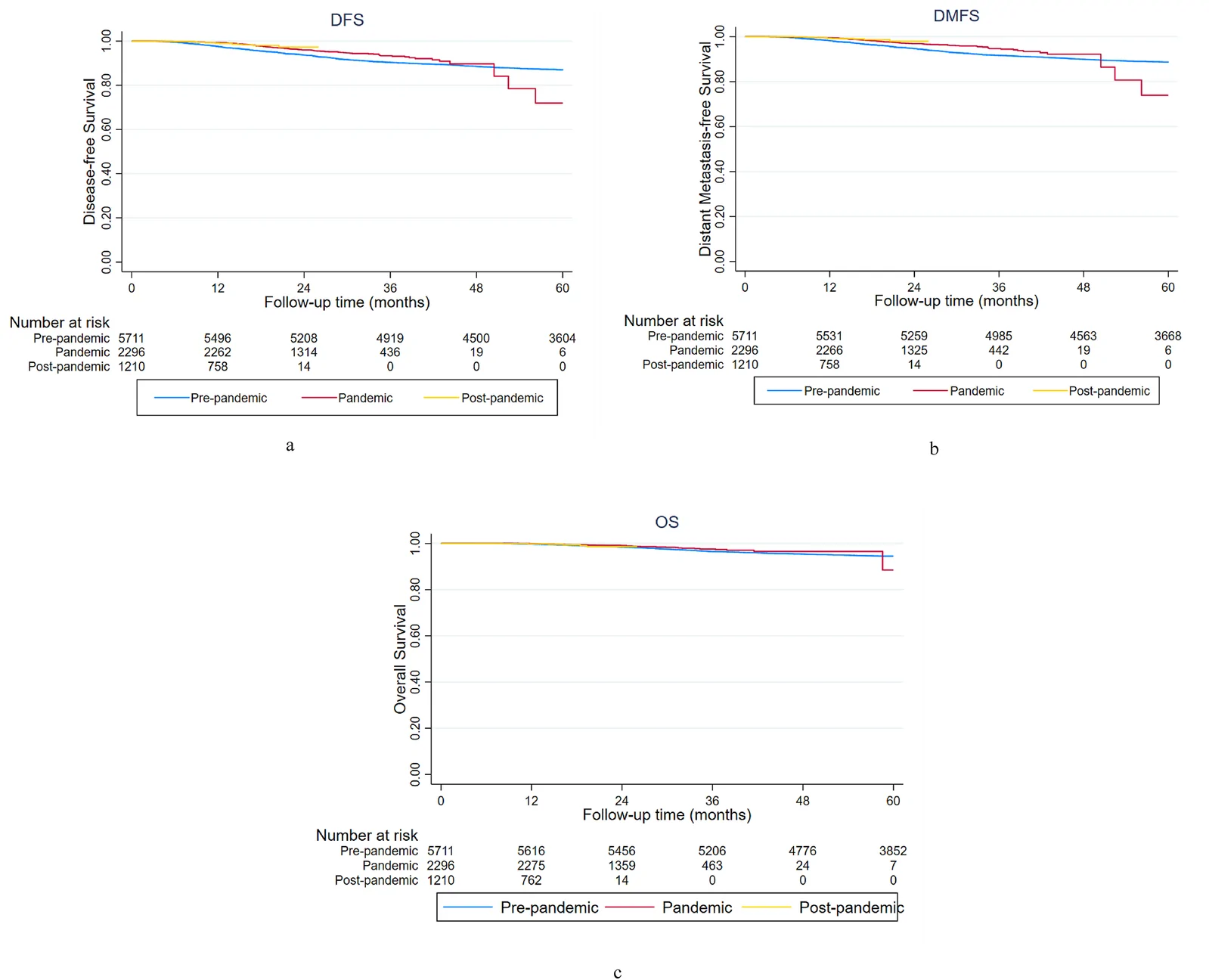
Kaplan-Meier survival curves stratified by COVID-19 periods (a, DFS, P < 0.001; b, DMFS, P < 0.001; c, OS, P = 0.215).

### 3.5 Sensitivity analyses

A comprehensive set of sensitivity analyses was performed to validate the robustness of the findings (Table 3).

**Table 3:** Sensitivity analyses for the primary analysis of other taxanes versus nab-paclitaxel in long-term survival.

Sensitivity analysis	DFS		DMFS		OS	
	HR (95% CI)	P	HR (95% CI)	P	HR (95% CI)	P
Overall PSM	1.165(0.913-1.486)	0.22	1.106(0.847-1.444)	0.46	1.068(0.713-1.599)	0.749
Overlap period restriction (2018–2019)	1.093(0.744-1.606)	0.65	0.978(0.64-1.494)	0.917	0.833(0.453-1.532)	0.556
Overlap-period PSM (2018–2019)	1.113(0.744-1.664)	0.602	1.066(0.680-1.671)	0.781	1.000(0.519-1.927)	1
Year as RCS	1.087(0.888-1.332)	0.419	0.984(0.791-1.224)	0.883	1.037(0.751-1.434)	0.824
Calendar-period stratified	1.064(0.863-1.311)	0.563	0.964(0.770-1.208)	0.751	1.023(0.734-1.426)	0.894
Multiple imputation (MI)	1.086(0.785-1.503)	0.6191	0.942(0.663-1.336)	0.7361	0.825(0.497-1.368)	0.456

Propensity Score Matching Analysis: 1:1 propensity score matching was performed, successfully matching 2542 patients (1271 per group). All covariates achieved balance with SMD <0.1 (Supplementary Table S6). Love plots and propensity score distribution plots are provided in Supplementary Fig. S2–S3. In the matched cohort, the stratified Cox regression showed no significant treatment effect for all endpoints: DFS (HR = 1.165, P = 0.220), DMFS (HR = 1.106, P = 0.460), and OS (HR = 1.068, P = 0.749).

Overlap-Period Restriction and PSM (2018–2019): Analysis restricted to the 2018–2019 overlap period showed no significant treatment effect for any endpoint: DFS HR = 1.093 (P = 0.650); DMFS HR = 0.978 (P = 0.917); OS HR = 0.833 (P = 0.556). PSM restricted to this overlap period (1056 matched patients) similarly showed no significant differences. Covariate balance after PSM in this overlap cohort is shown in Supplementary Fig. S4–S5 and Supplementary Table S7.

Year of Diagnosis as Restricted Cubic Splines: Allowing flexible non-linear adjustment for calendar year, the treatment effect remained non-significant for all endpoints: DFS HR = 1.087 (P = 0.419); DMFS HR = 0.984 (P = 0.883); OS HR = 1.037 (P = 0.824). Non-linearity tests were not significant for any endpoint (DFS P = 0.337; DMFS P = 0.099; OS P = 0.109).

Calendar-Period Stratified Analysis: Stratifying by calendar period, the treatment effect remained non-significant: DFS HR = 1.064 (P = 0.563); DMFS HR = 0.964 (P = 0.751); OS HR = 1.023 (P = 0.894).

Multiple Imputation: After multiple imputation for missing covariates, the treatment effect remained non-significant for all endpoints: DFS HR = 1.086 (P = 0.619); DMFS HR = 0.942 (P = 0.736); OS HR = 0.825 (P = 0.456).

### 3.6 Subgroup analysis

Interaction analyses were performed in the whole population to examine whether the association between taxane type and outcomes varied across clinical characteristics. For DFS, significant interactions were observed between drug type and cN stage, immunohistochemical subtype, axillary surgery type, endocrine therapy, HER2-targeted therapy, and radiotherapy (all P for interaction <0.05). For DMFS, significant interactions were observed with cN stage, immunohistochemical subtype, axillary surgery type, endocrine therapy, and HER2-targeted therapy (all P for interaction <0.05). For OS, significant interactions were observed with WHO grade, immunohistochemical subtype, endocrine therapy, and radiotherapy (all P for interaction <0.05). Subgroup estimates are presented in Forest plots (Supplementary Fig. S6–S8) and Supplementary Table S8–S10.

### 3.7 Neoadjuvant chemotherapy subgroup: pathological complete response

In the neoadjuvant chemotherapy subgroup, the pCR rate was significantly higher in the nab-paclitaxel group than in the other taxanes group (37.6% vs. 18.6%). After adjusting for clinicopathological covariates (Model 1), the OR for nab-paclitaxel vs. other taxanes was 2.985 (95% CI 2.500–3.559, P < 0.001). After additional adjustment for year of diagnosis (Model 2), the OR was 1.529 (95% CI 1.214–1.927, P < 0.001). After adjustment for COVID-19 periods (Model 3), the OR was 1.946 (95% CI 1.553–2.433, P < 0.001).

## 4 Discussion

Based on real-world data from 9217 patients with early breast cancer, this study compared the long-term outcomes of nab-paclitaxel versus other taxanes. The primary analysis, adjusting for year of diagnosis as a categorical variable, demonstrated that the apparent DFS and DMFS advantages of nab-paclitaxel observed without time adjustment were no longer statistically significant after accounting for calendar-time confounding. This finding was consistently supported by all sensitivity analyses. In a complementary analysis adjusting for COVID-19 periods, a DFS finding was observed. However, this adjustment captures pandemic-related disruptions rather than the long-term secular trends in breast cancer care that constitute the core of calendar-time bias. As such, this finding does not alter the primary conclusion that no significant treatment effect was observed after appropriate adjustment for calendar-time confounding.

Calendar-time bias is an increasingly recognized methodological issue in long-term observational oncology research [[11], [12], [13]]. In this study, the distribution of year of diagnosis was imbalanced between the two groups: nab-paclitaxel was predominantly used in recent years, whereas other taxanes were mostly used in earlier periods. Over recent years, the overall standard of breast cancer treatment has improved, including the widespread adoption of targeted therapies, optimization of supportive treatment, maturation of multidisciplinary team approaches, and enhancement of follow-up systems – all of which are associated with patient outcomes [14]. The increase in the use of nab-paclitaxel at our center coincided with the implementation of the national volume-based drug procurement policy, which reduced drug prices and improved accessibility [15,16]. Thus, treatment choice is influenced not only by clinical factors but also by healthcare policies and drug accessibility, leading to an association between treatment assignment and treatment era [17]. Failure to adjust for this temporal trend may cause observed differences in outcomes to reflect improvements in survival due to advances in medical care rather than differences in drug efficacy.

Previous randomized controlled trials comparing nab-paclitaxel with conventional taxanes in early breast cancer have yielded inconsistent results. The GeparSepto study showed that nab-paclitaxel improved the pathological complete response rate compared with solvent-based paclitaxel in the neoadjuvant setting, particularly in triple-negative breast cancer, but long-term survival benefits were not consistently demonstrated across all endpoints [8]. The ETNA study found no significant difference in event-free survival between the two paclitaxel regimens as neoadjuvant therapy for HER2-negative breast cancer [18]. In the adjuvant setting, one study reported no significant difference in DFS between nab-paclitaxel and solvent-based paclitaxel [19]. Some studies have suggested a potential advantage of nab-paclitaxel in the HER2-positive subgroup, but these findings are largely exploratory [20]. Overall, existing randomized evidence suggests that nab-paclitaxel may offer advantages in short-term efficacy measures, but its long-term survival benefit remains uncertain. In our neoadjuvant subgroup, nab-paclitaxel was associated with significantly higher pCR rates compared with other taxanes; however, this short-term pathological benefit did not translate into a significant long-term survival advantage after adjustment for calendar-time confounding, consistent with the pattern observed in previous studies where improved pCR rates did not always correlate with improved DFS or OS [9]. Because the true efficacy difference may be small, real-world studies are susceptible to calendar-time confounding, which can produce apparent treatment differences. Our results are consistent with this evidence: after adjustment for temporal factors using the year-adjusted model, the long-term survival outcomes of the two taxanes were not significantly different.

A key methodological consideration is the distinction between two levels of time effects. Year of diagnosis, categorized into four periods, captures the cumulative long-term secular advances in breast cancer care, whereas COVID-19 period classification captures the acute disruption of a specific historical event. Because the 2020–2024 period in the year categorization encompasses both the pandemic and post-pandemic periods defined in the COVID-19 classification, this overlap creates collinearity that precludes simultaneous adjustment in a single Cox model. We therefore adopted the year-adjusted model as the primary analytical framework, as it most directly captures the secular trend central to calendar-time bias, and included the COVID-19-adjusted model as a complementary analysis. The use of categorical year adjustment, rather than assuming a linear relationship, allows for more flexible adjustment for calendar time over a 17-year period. After propensity score matching—with both year of diagnosis and COVID-19 period included as matching variables—all covariates achieved balance, suggesting that in long-term real-world comparative studies, time variables should be adjusted for as covariates in regression models in addition to or instead of relying solely on matching methods [[21], [22], [23]]. The survival curves stratified by COVID-19 period further illustrate this complexity, showing that different temporal mechanisms can exert opposing effects.

Beyond this methodological framework, the observed prognostic impact of time was also notable. Year of diagnosis emerged as an independent prognostic factor: later diagnosis was associated with better DFS, DMFS, and OS, reflecting improvements in breast cancer care over time. The pandemic added further complexity—patients treated during the pandemic may have faced treatment delays or changes in healthcare resource allocation [[24], [25], [26]], and in the COVID-19-adjusted model, the post-pandemic period was associated with better DFS and DMFS, suggesting that recovery of healthcare services and continued therapeutic advances may have contributed to improved outcomes.

This study has several limitations. First, as a single-center retrospective analysis, despite the use of multiple methods to control for confounding, residual confounding cannot be completely excluded. Second, because nab-paclitaxel has been widely used only in recent years, its follow-up duration is shorter than that of the other taxanes group, which may limit the ability to detect long-term survival differences. Third, the comparator group was heterogeneous, as it included both docetaxel and solvent-based paclitaxel, though the former comprised the vast majority. Fourth, this study did not control for factors such as dose density of different taxanes, combination regimens, and treatment sequence. Fifth, missing data were present for several covariates. While we treated missing values as a separate category in the primary analysis and performed multiple imputation as a sensitivity analysis, the non-random nature of missingness may introduce residual confounding that cannot be fully adjusted for. In addition, the subgroup analyses are exploratory, and some subgroups had small numbers of events, warranting cautious interpretation.

In conclusion, after adjustment for calendar-time confounding using categorical year adjustment, no significant difference in long-term survival outcomes was observed between nab-paclitaxel and other taxanes in patients with early breast cancer. The absence of a significant difference should not be interpreted as evidence of equivalence or non-inferiority, as the study was not designed to test these hypotheses. This study demonstrates that in long-term real-world comparative studies, calendar-time bias can produce spurious treatment effects. Future evaluations of treatment strategies that are adopted over time should carefully consider and adjust for calendar-time confounding.

## CRediT authorship contribution statement

**Jingjing Xiao:** Writing – original draft, Formal analysis, Data curation, Conceptualization. **Meiling Huang:** Validation, Investigation. **Changjiao Yan:** Visualization, Software. **Zhe Wang:** Writing – review & editing, Methodology. **Ting Wang:** Supervision, Project administration.

## Informed consent

The Ethics Committee of Xijing Hospital approved a waiver of informed consent for this retrospective study, as the data were analyzed anonymously and no additional interventions were performed beyond routine clinical practice.

## Data availability

The datasets generated during and/or analyzed during the current study are available from the corresponding author on reasonable request.

## Ethical approval

All procedures performed in studies involving human participants were in accordance with the ethical standards of the institutional and national research committee and with the 1964 Helsinki declaration and its later amendments or comparable ethical standards.

This article does not contain any studies with animals performed by any of the authors.

## Declaration of competing interest

The authors declare that they have no known competing financial interests or personal relationships that could have appeared to influence the work reported in this paper.
