# Supplementary material for: Analysis of outcomes of nab-paclitaxel compared to other taxanes in early breast cancer: A study revealing the critical role of calendar-time bias in real-world drug comparisons
Source: Breast. 2026 Sep 25;90:104934. doi: 10.1016/j.breast.2026.104934 (PMC13651569; doi:10.1016/j.breast.2026.104934)
Supplement: Supplementary file 1 [file mmc1.docx]

Supplementary Figure S1. Flowchart of patient data screening and selection.

Excluded:

- Non-invasive breast cancer (n=912)
- Male (n=69)
- Age＜18 (n=3)
- M1 (n=679)
- Bilateral breast cancer (n=177)
- Inflammatory breast cancer (n=3)
- No chemotherapy information (n=1723)
- No surgery information (n=481)
- No taxane use (n=476)
- Taxane cycles < 4 (n=318)

Diagnosed with breast cancer from 2007-01-01 to 2024-07-31 (n=14058)

Eligible and in analysis (n=9217)

Nab-paclitaxel group (n=3860)

Other taxanes group (n=5357)

Supplementary Figure S2. Love plot showing standardized mean differences before and after PSM (overall cohort).

Supplementary Figure S3. Propensity score distribution before and after PSM (overall cohort).

Supplementary Figure S4. Love plot showing standardized mean differences before and after PSM in the overlap cohort (2018–2019).

Supplementary Figure S5. Propensity score distribution before and after PSM in the overlap cohort (2018–2019).

Supplementary Figures S6. Forest plot of subgroup analyses for DFS.

Supplementary Figures S7. Forest plot of subgroup analyses for DMFS.

Supplementary Figures S8. Forest plot of subgroup analyses for OS.

| Endpoint | Treatment Group | N | Events, n (%) | 3-year | | 5-year | | 10-year | |
| --- | --- | --- | --- | --- | --- | --- | --- | --- | --- |
|  |  |  |  | Survival Rate (95% CI) | P | Survival Rate (95% CI) | P | Survival Rate (95% CI) | P |
| DFS | Nab-paclitaxel | 3860 | 211(5.5) | 93.2 (92.2–94.2) | <0.001 | 89.3 (87.3–91.3) | 0.025 | 79.5 (74.2–84.8) | 1 |
|  | Other taxanes | 5357 | 867(16.2) | 90.2 (89.4–91.0) |  | 86.8 (85.8–87.8) |  | 79.5 (78.1–80.9) |  |
|  | Overall | 9217 | 1078(11.7) | 91.3 |  | 87.8 |  | 80.2 |  |
| DMFS | Nab-paclitaxel | 3860 | 179(4.6) | 94.3 (93.3–95.3) | <0.001 | 91.1 (89.3–92.9) | 0.012 | 79.9 (74.4–85.4) | 0.386 |
|  | Other taxanes | 5357 | 742(13.9) | 91.6 (90.8–92.4) |  | 88.5 (87.5–89.5) |  | 82.4 (81.0–83.8) |  |
|  | Overall | 9217 | 921(10.0) | 92.5 |  | 89.4 |  | 82.9 |  |
| OS | Nab-paclitaxel | 3860 | 78(2.0) | 97.3 (96.5–98.1) | 0.072 | 95.5 (94.1–96.9) | 0.115 | 91.1 (88.2–94.0) | 0.95 |
|  | Other taxanes | 5357 | 361(6.7) | 96.4 (95.8–97.0) |  | 94.3 (93.7–94.9) |  | 91.2 (90.2–92.2) |  |
|  | Overall | 9217 | 439(4.8) | 96.7 |  | 94.7 |  | 91.5 |  |

Supplementary Table S1. Survival Rates by Treatment Group and Time Point.

|  |  | Total | | Nab-paclitaxel | | Other taxanes | | P |
| --- | --- | --- | --- | --- | --- | --- | --- | --- |
|  |  | N（9217） | % | N（3860） | % | N（5357） | % |  |
| Year of diagnosis | 2007 | 51 | 0.6% | 6a | 11.8% | 45b | 88.2% | ＜0.001 |
|  | 2008 | 159 | 1.7% | 11a | 6.9% | 148b | 93.1% |  |
|  | 2009 | 229 | 2.5% | 14a | 6.1% | 215b | 93.9% |  |
|  | 2010 | 299 | 3.2% | 33a | 11.0% | 266b | 89.0% |  |
|  | 2011 | 379 | 4.1% | 20a | 5.3% | 359b | 94.7% |  |
|  | 2012 | 383 | 4.2% | 18a | 4.7% | 365b | 95.3% |  |
|  | 2013 | 458 | 5.0% | 43a | 9.4% | 415b | 90.6% |  |
|  | 2014 | 566 | 6.1% | 29a | 5.1% | 537b | 94.9% |  |
|  | 2015 | 562 | 6.1% | 7a | 1.2% | 555b | 98.8% |  |
|  | 2016 | 580 | 6.3% | 10a | 1.7% | 570b | 98.3% |  |
|  | 2017 | 658 | 7.1% | 41a | 6.2% | 617b | 93.8% |  |
|  | 2018 | 647 | 7.0% | 164a | 25.3% | 483b | 74.7% |  |
|  | 2019 | 740 | 8.0% | 454a | 61.4% | 286b | 38.6% |  |
|  | 2020 | 615 | 6.7% | 507a | 82.4% | 108b | 17.6% |  |
|  | 2021 | 723 | 7.8% | 602a | 83.3% | 121b | 16.7% |  |
|  | 2022 | 726 | 7.9% | 621a | 85.5% | 105b | 14.5% |  |
|  | 2023 | 921 | 10.0% | 822a | 89.3% | 99b | 10.7% |  |
|  | 2024 | 521 | 5.7% | 458a | 87.9% | 63b | 12.1% |  |

Supplementary Table S2. Annual distribution of diagnosis year by treatment group.

|  |  | DFS | | DMFS | | OS | |
| --- | --- | --- | --- | --- | --- | --- | --- |
|  |  | P | HR ( 95% CI ) | P | HR ( 95% CI ) | P | HR ( 95% CI ) |
| Age | ≤35 | ＜0.001 | 1 | ＜0.001 | 1 | ＜0.001 | 1 |
|  | 36-45 | ＜0.001 | 0.642(0.529-0.779) | ＜0.001 | 0.612(0.497-0.755) | 0.001 | 0.593(0.436-0.807) |
|  | 46-55 | ＜0.001 | 0.623(0.515-0.754) | ＜0.001 | 0.6(0.489-0.737) | ＜0.001 | 0.507(0.372-0.689) |
|  | 56-69 | ＜0.001 | 0.691(0.562-0.851) | ＜0.001 | 0.646(0.517-0.808) | 0.11 | 0.774(0.565-1.06) |
|  | ≥70 | 0.623 | 0.874(0.51-1.496) | 0.152 | 0.644(0.353-1.176) | 0.929 | 0.969(0.485-1.936) |
| cT | 0 | ＜0.001 | 1 | ＜0.001 | 1 | ＜0.001 | 1 |
|  | 1 | 0.683 | 1.338(0.331-5.415) | 0.796 | 1.203(0.297-4.878) | — | — |
|  | 2 | 0.452 | 1.707(0.424-6.87) | 0.555 | 1.522(0.378-6.131) | — | — |
|  | 3 | 0.286 | 2.146(0.527-8.732) | 0.369 | 1.905(0.467-7.768) | — | — |
|  | 4 | 0.099 | 3.266(0.799-13.35) | 0.143 | 2.868(0.7-11.748) | — | — |
|  | Unknown | 0.055 | 4.033(0.971-16.741) | 0.15 | 2.868(0.683-12.052) | — | — |
| cN | 0 | ＜0.001 | 1 | ＜0.001 | 1 | ＜0.001 | 1 |
|  | 1 | ＜0.001 | 1.413(1.208-1.652) | ＜0.001 | 1.519(1.279-1.805) | ＜0.001 | 1.684(1.309-2.167) |
|  | 2 | ＜0.001 | 2.317(1.761-3.049) | ＜0.001 | 2.52(1.892-3.357) | ＜0.001 | 2.763(1.865-4.094) |
|  | 3 | ＜0.001 | 3.059(2.35-3.981) | ＜0.001 | 3.247(2.453-4.298) | ＜0.001 | 2.924(1.942-4.403) |
|  | Unknown | ＜0.001 | 4.961(2.841-8.662) | ＜0.001 | 4.782(2.469-9.26) | 0.02 | 4.443(1.27-15.544) |
| WHO grade | 1 | ＜0.001 | 1 | ＜0.001 | 1 | ＜0.001 | 1 |
|  | 2 | 0.011 | 1.792(1.141-2.816) | 0.019 | 1.827(1.103-3.026) | 0.036 | 2.916(1.075-7.91) |
|  | 3 | 0.001 | 2.283(1.43-3.645) | 0.001 | 2.412(1.433-4.061) | 0.003 | 4.598(1.671-12.652) |
|  | Unknown | 0.049 | 1.587(1.001-2.516) | 0.037 | 1.727(1.034-2.884) | 0.02 | 3.296(1.207-9.001) |
| Ki-67 | ≤14% | 0.262 | 1 | 0.319 | 1 | 0.412 | 1 |
|  | 15%-50% | 0.966 | 0.992(0.701-1.405) | 0.996 | 1.001(0.688-1.456) | 0.97 | 1.011(0.58-1.76) |
|  | ＞50% | 0.439 | 1.153(0.804-1.653) | 0.426 | 1.171(0.794-1.727) | 0.481 | 1.228(0.694-2.174) |
|  | Unknown | 0.491 | 1.148(0.776-1.698) | 0.569 | 1.131(0.741-1.725) | 0.538 | 1.215(0.655-2.254) |
| Immunohistochemical subtyping | Luminal A | ＜0.001 | 1 | 0.001 | 1 | 0.035 | 1 |
|  | Luminal B | 0.016 | 1.712(1.106-2.653) | 0.019 | 1.77(1.099-2.852) | 0.519 | 1.256(0.628-2.508) |
|  | HER2+(HR-) | 0.003 | 3.302(1.505-7.243) | 0.002 | 3.837(1.641-8.976) | 0.049 | 3.026(1.005-9.106) |
|  | HER2+(HR+) | 0.001 | 2.747(1.496-5.045) | 0.001 | 2.964(1.542-5.698) | 0.031 | 2.821(1.102-7.223) |
|  | Triple-negative | 0.121 | 1.791(0.858-3.742) | 0.074 | 2.07(0.933-4.594) | 0.829 | 1.122(0.394-3.198) |
|  | Unknown | 0.132 | 1.509(0.883-2.577) | 0.072 | 1.7(0.955-3.028) | 0.364 | 1.464(0.643-3.335) |
| Breast surgery type | Breast-conserving surgery | ＜0.001 | 1 | ＜0.001 | 1 | ＜0.001 | 1 |
|  | Mastectomy | ＜0.001 | 1.553(1.283-1.88) | ＜0.001 | 1.795(1.448-2.226) | ＜0.001 | 2.254(1.593-3.189) |
|  | Mastectomy with reconstruction | 0.632 | 1.08(0.788-1.481) | 0.63 | 0.911(0.622-1.333) | 0.68 | 0.875(0.463-1.652) |
| Axillary surgery type | Sentinel lymph node biopsy | — | 1 | — | 1 | — | 1 |
|  | Axillary lymph node dissection | ＜0.001 | 1.524(1.211-1.919) | ＜0.001 | 2.099(1.572-2.803) | 0.002 | 2.006(1.293-3.111) |
| Neoadjuvant chemotherapy | Yes | — | 1 | — | 1 | — | 1 |
|  | No | ＜0.001 | 0.701(0.609-0.808) | ＜0.001 | 0.666(0.571-0.776) | 0.001 | 0.681(0.546-0.85) |
| Endocrine therapy | Yes | 0.099 | 1 | 0.027 | 1 | ＜0.001 | 1 |
|  | No | 0.373 | 1.321(0.716-2.437) | 0.618 | 1.184(0.61-2.295) | 0.03 | 2.513(1.091-5.785) |
|  | Unknown | 0.039 | 1.178(1.008-1.377) | 0.007 | 1.258(1.064-1.486) | ＜0.001 | 2.079(1.603-2.698) |
| Radiotherapy | Yes | — | 1 | — | 1 | — | 1 |
|  | Unknown | 0.002 | 0.801(0.696-0.921) | 0.001 | 0.77(0.662-0.895) | 0.05 | 0.806(0.65-1) |
| HER2-targeted therapy | Yes | ＜0.001 | 1 | ＜0.001 | 1 | ＜0.001 | 1 |
|  | No | ＜0.001 | 2.687(1.701-4.244) | ＜0.001 | 2.822(1.734-4.592) | ＜0.001 | 5.066(2.51-10.227) |
|  | Unknown | ＜0.001 | 1.9(1.508-2.394) | ＜0.001 | 1.989(1.551-2.55) | ＜0.001 | 2.836(1.971-4.081) |
| Taxane type | Nab-paclitaxel | — | 1 | — | 1 | — | 1 |
|  | Other taxanes | ＜0.001 | 1.366(1.16-1.608) | 0.004 | 1.294(1.084-1.544) | 0.532 | 1.087(0.836-1.414) |

Supplementary Table S3. Complete Model 1 Cox regression results (all covariates).

|  |  | DFS | | DMFS | | OS | |
| --- | --- | --- | --- | --- | --- | --- | --- |
|  |  | P | HR ( 95% CI ) | P | HR ( 95% CI ) | P | HR ( 95% CI ) |
| Age | ≤35 | ＜0.001 | 1 | ＜0.001 | 1 | ＜0.001 | 1 |
|  | 36-45 | ＜0.001 | 0.641(0.529-0.778) | ＜0.001 | 0.614(0.498-0.756) | 0.001 | 0.596(0.438-0.81) |
|  | 46-55 | ＜0.001 | 0.631(0.521-0.764) | ＜0.001 | 0.609(0.496-0.749) | ＜0.001 | 0.517(0.38-0.704) |
|  | 56-69 | 0.001 | 0.708(0.575-0.871) | ＜0.001 | 0.666(0.533-0.832) | 0.175 | 0.804(0.587-1.102) |
|  | ≥70 | 0.687 | 0.895(0.522-1.534) | 0.206 | 0.678(0.372-1.238) | 0.928 | 1.032(0.519-2.055) |
| cT | 0 | ＜0.001 | 1 | ＜0.001 | 1 | ＜0.001 | 1 |
|  | 1 | 0.767 | 1.235(0.305-5.002) | 0.893 | 1.101(0.271-4.467) | — | — |
|  | 2 | 0.516 | 1.588(0.394-6.396) | 0.631 | 1.407(0.349-5.673) | — | — |
|  | 3 | 0.298 | 2.107(0.517-8.578) | 0.384 | 1.867(0.458-7.621) | — | — |
|  | 4 | 0.117 | 3.086(0.754-12.624) | 0.168 | 2.696(0.658-11.052) | — | — |
|  | Unknown | 0.066 | 3.809(0.917-15.812) | 0.175 | 2.7(0.642-11.346) | — | — |
| cN | 0 | ＜0.001 | 1 | ＜0.001 | 1 | ＜0.001 | 1 |
|  | 1 | ＜0.001 | 1.448(1.238-1.694) | ＜0.001 | 1.56(1.313-1.854) | ＜0.001 | 1.767(1.373-2.273) |
|  | 2 | ＜0.001 | 2.377(1.806-3.128) | ＜0.001 | 2.601(1.952-3.466) | ＜0.001 | 3.061(2.066-4.535) |
|  | 3 | ＜0.001 | 3.436(2.632-4.485) | ＜0.001 | 3.686(2.775-4.896) | ＜0.001 | 3.43(2.268-5.186) |
|  | Unknown | ＜0.001 | 5.343(3.063-9.322) | ＜0.001 | 5.18(2.678-10.017) | 0.013 | 4.842(1.393-16.823) |
| WHO grade | 1 | ＜0.001 | 1 | ＜0.001 | 1 | 0.001 | 1 |
|  | 2 | 0.02 | 1.713(1.09-2.691) | 0.033 | 1.732(1.045-2.868) | 0.048 | 2.732(1.007-7.411) |
|  | 3 | 0.001 | 2.177(1.364-3.476) | 0.002 | 2.275(1.352-3.83) | 0.005 | 4.227(1.536-11.627) |
|  | Unknown | 0.093 | 1.485(0.936-2.354) | 0.074 | 1.596(0.955-2.666) | 0.027 | 3.097(1.134-8.459) |
| Ki-67 | ≤14% | 0.232 | 1 | 0.234 | 1 | 0.247 | 1 |
|  | 15%-50% | 0.846 | 1.035(0.731-1.466) | 0.792 | 1.052(0.723-1.531) | 0.703 | 1.114(0.64-1.941) |
|  | ＞50% | 0.279 | 1.221(0.851-1.752) | 0.26 | 1.251(0.847-1.847) | 0.243 | 1.406(0.793-2.493) |
|  | Unknown | 0.768 | 1.061(0.716-1.572) | 0.834 | 1.046(0.685-1.598) | 0.835 | 1.068(0.576-1.982) |
| Immunohistochemical subtyping | Luminal A | ＜0.001 | 1 | 0.001 | 1 | 0.007 | 1 |
|  | Luminal B | 0.018 | 1.693(1.093-2.623) | 0.022 | 1.747(1.084-2.814) | 0.569 | 1.223(0.612-2.441) |
|  | HER2+(HR-) | 0.002 | 3.42(1.557-7.511) | 0.002 | 3.963(1.692-9.283) | 0.04 | 3.168(1.051-9.543) |
|  | HER2+(HR+) | 0.002 | 2.651(1.443-4.872) | 0.002 | 2.833(1.473-5.447) | 0.031 | 2.829(1.102-7.263) |
|  | Triple-negative | 0.099 | 1.861(0.889-3.892) | 0.059 | 2.157(0.97-4.797) | 0.83 | 1.122(0.393-3.204) |
|  | Unknown | 0.204 | 1.416(0.828-2.421) | 0.12 | 1.582(0.887-2.821) | 0.58 | 1.262(0.554-2.879) |
| Breast surgery type | Breast-conserving surgery | ＜0.001 | 1 | ＜0.001 | 1 | ＜0.001 | 1 |
|  | Mastectomy | ＜0.001 | 1.534(1.267-1.858) | ＜0.001 | 1.768(1.426-2.194) | ＜0.001 | 2.155(1.523-3.049) |
|  | Mastectomy with reconstruction | 0.421 | 1.139(0.83-1.563) | 0.866 | 0.968(0.66-1.418) | 0.841 | 0.937(0.495-1.772) |
| Axillary surgery type | Sentinel lymph node biopsy | — | 1 | — | 1 | — | 1 |
|  | Axillary lymph node dissection | 0.012 | 1.349(1.068-1.706) | ＜0.001 | 1.84(1.373-2.465) | 0.024 | 1.676(1.072-2.62) |
| Neoadjuvant chemotherapy | Yes | — | 1 | — | 1 | — | 1 |
|  | No | ＜0.001 | 0.691(0.6-0.796) | ＜0.001 | 0.655(0.562-0.763) | ＜0.001 | 0.669(0.536-0.833) |
| Endocrine therapy | Yes | 0.081 | 1 | 0.018 | 1 | ＜0.001 | 1 |
|  | No | 0.498 | 1.236(0.67-2.281) | 0.778 | 1.1(0.567-2.134) | 0.039 | 2.408(1.045-5.549) |
|  | Unknown | 0.027 | 1.193(1.02-1.395) | 0.004 | 1.275(1.078-1.507) | ＜0.001 | 2.174(1.674-2.824) |
| Radiotherapy | Yes | — | 1 | — | 1 | — | 1 |
|  | Unknown | 0.012 | 0.834(0.724-0.961) | 0.006 | 0.806(0.692-0.939) | 0.119 | 0.842(0.679-1.045) |
| HER2-targeted therapy | Yes | ＜0.001 | 1 | ＜0.001 | 1 | ＜0.001 | 1 |
|  | No | ＜0.001 | 2.505(1.584-3.963) | ＜0.001 | 2.591(1.59-4.222) | ＜0.001 | 4.845(2.391-9.816) |
|  | Unknown | ＜0.001 | 1.696(1.341-2.144) | ＜0.001 | 1.751(1.36-2.253) | ＜0.001 | 2.447(1.687-3.55) |
| Taxane type | Nab-paclitaxel | — | 1 | — | 1 | — | 1 |
|  | Other taxanes | 0.536 | 1.068(0.867-1.316) | 0.789 | 0.97(0.775-1.214) | 0.932 | 1.015(0.729-1.412) |
| Year of diagnosis | 2007-2012 | ＜0.001 | 1 | ＜0.001 | 1 | ＜0.001 | 1 |
|  | 2013-2017 | 0.001 | 0.768(0.655-0.901) | 0.002 | 0.764(0.644-0.905) | ＜0.001 | 0.52(0.409-0.661) |
|  | 2018-2019 | ＜0.001 | 0.503(0.392-0.645) | ＜0.001 | 0.471(0.359-0.617) | ＜0.001 | 0.421(0.287-0.617) |
|  | 2020-2024 | ＜0.001 | 0.528(0.392-0.71) | ＜0.001 | 0.487(0.352-0.673) | 0.028 | 0.583(0.361-0.943) |

Supplementary Table S4. Complete Model 2 Cox regression results (all covariates).

|  |  | OS | | DFS | | DMFS | |
| --- | --- | --- | --- | --- | --- | --- | --- |
|  |  | P | HR ( 95% CI ) | P | HR ( 95% CI ) | P | HR ( 95% CI ) |
| Age | ≤35 | ＜0.001 | 1 | ＜0.001 | 1 | ＜0.001 | 1 |
|  | 36-45 | 0.001 | 0.592(0.436-0.806) | ＜0.001 | 0.644(0.53-0.781) | ＜0.001 | 0.614(0.498-0.756) |
|  | 46-55 | ＜0.001 | 0.505(0.371-0.687) | ＜0.001 | 0.625(0.516-0.757) | ＜0.001 | 0.603(0.491-0.74) |
|  | 56-69 | 0.104 | 0.77(0.562-1.055) | 0.001 | 0.696(0.565-0.857) | ＜0.001 | 0.652(0.522-0.815) |
|  | ≥70 | 0.914 | 0.963(0.482-1.924) | 0.649 | 0.882(0.515-1.512) | 0.163 | 0.651(0.357-1.189) |
| cT | 0 | ＜0.001 | 1 | ＜0.001 | 1 | ＜0.001 | 1 |
|  | 1 | — | — | 0.691 | 1.328(0.328-5.377) | 0.806 | 1.191(0.294-4.832) |
|  | 2 | — | — | 0.461 | 1.689(0.419-6.8) | 0.567 | 1.502(0.373-6.054) |
|  | 3 | — | — | 0.292 | 2.128(0.523-8.663) | 0.377 | 1.885(0.462-7.691) |
|  | 4 | — | — | 0.103 | 3.23(0.79-13.208) | 0.148 | 2.831(0.691-11.597) |
|  | Unknown | — | — | 0.054 | 4.042(0.973-16.784) | 0.149 | 2.876(0.684-12.088) |
| cN | 0 | ＜0.001 | 1 | ＜0.001 | 1 | ＜0.001 | 1 |
|  | 1 | ＜0.001 | 1.68(1.305-2.162) | ＜0.001 | 1.419(1.213-1.659) | ＜0.001 | 1.529(1.287-1.816) |
|  | 2 | ＜0.001 | 2.773(1.871-4.109) | ＜0.001 | 2.305(1.752-3.033) | ＜0.001 | 2.507(1.882-3.34) |
|  | 3 | ＜0.001 | 2.914(1.935-4.388) | ＜0.001 | 3.07(2.359-3.997) | ＜0.001 | 3.264(2.465-4.322) |
|  | Unknown | 0.02 | 4.436(1.267-15.524) | ＜0.001 | 4.981(2.852-8.699) | ＜0.001 | 4.807(2.481-9.31) |
| WHO grade | 1 | ＜0.001 | 1 | ＜0.001 | 1 | ＜0.001 | 1 |
|  | 2 | 0.035 | 2.92(1.077-7.921) | 0.011 | 1.798(1.145-2.826) | 0.019 | 1.832(1.106-3.034) |
|  | 3 | 0.003 | 4.605(1.674-12.672) | 0.001 | 2.288(1.433-3.652) | 0.001 | 2.414(1.434-4.064) |
|  | Unknown | 0.019 | 3.324(1.217-9.08) | 0.055 | 1.569(0.99-2.488) | 0.042 | 1.702(1.019-2.844) |
| Ki-67 | ≤14% | 0.419 | 1 | 0.238 | 1 | 0.29 | 1 |
|  | 15%-50% | 0.966 | 1.012(0.581-1.762) | 0.968 | 0.993(0.701-1.405) | 0.992 | 1.002(0.689-1.458) |
|  | ＞50% | 0.482 | 1.227(0.693-2.172) | 0.423 | 1.159(0.808-1.661) | 0.407 | 1.179(0.799-1.738) |
|  | Unknown | 0.533 | 1.217(0.656-2.259) | 0.484 | 1.15(0.777-1.702) | 0.558 | 1.135(0.744-1.731) |
| Immunohistochemical subtyping | Luminal A | 0.034 | 1 | ＜0.001 | 1 | 0.001 | 1 |
|  | Luminal B | 0.526 | 1.251(0.626-2.499) | 0.015 | 1.718(1.109-2.661) | 0.018 | 1.777(1.103-2.864) |
|  | HER2+(HR-) | 0.049 | 3.022(1.004-9.094) | 0.003 | 3.316(1.512-7.275) | 0.002 | 3.85(1.646-9.005) |
|  | HER2+(HR+) | 0.031 | 2.823(1.102-7.23) | 0.001 | 2.739(1.491-5.031) | 0.001 | 2.953(1.536-5.676) |
|  | Triple-negative | 0.836 | 1.117(0.392-3.182) | 0.118 | 1.799(0.861-3.758) | 0.072 | 2.078(0.936-4.614) |
|  | Unknown | 0.364 | 1.464(0.643-3.334) | 0.133 | 1.507(0.882-2.574) | 0.073 | 1.696(0.952-3.021) |
| Breast surgery type | Breast-conserving surgery | ＜0.001 | 1 | ＜0.001 | 1 | ＜0.001 | 1 |
|  | Mastectomy | ＜0.001 | 2.255(1.594-3.19) | ＜0.001 | 1.555(1.284-1.882) | ＜0.001 | 1.795(1.447-2.226) |
|  | Mastectomy with reconstruction | 0.67 | 0.871(0.461-1.645) | 0.571 | 1.095(0.799-1.502) | 0.685 | 0.924(0.631-1.353) |
| Axillary surgery type | Sentinel lymph node biopsy | — | 1 | — | 1 | — | 1 |
|  | Axillary lymph node dissection | 0.002 | 2.025(1.304-3.145) | 0.001 | 1.498(1.189-1.886) | ＜0.001 | 2.056(1.539-2.746) |
| Neoadjuvant chemotherapy | Yes | — | 1 | — | 1 | — | 1 |
|  | No | 0.001 | 0.682(0.547-0.852) | ＜0.001 | 0.698(0.605-0.804) | ＜0.001 | 0.662(0.568-0.772) |
| Endocrine therapy | Yes | ＜0.001 | 1 | 0.083 | 1 | 0.021 | 1 |
|  | No | 0.03 | 2.52(1.094-5.803) | 0.378 | 1.317(0.714-2.429) | 0.624 | 1.18(0.609-2.288) |
|  | Unknown | ＜0.001 | 2.078(1.601-2.697) | 0.031 | 1.187(1.016-1.388) | 0.006 | 1.268(1.072-1.499) |
| Radiotherapy | Yes | — | 1 | — | 1 | — | 1 |
|  | Unknown | 0.044 | 0.801(0.645-0.994) | 0.003 | 0.806(0.7-0.928) | 0.001 | 0.777(0.667-0.905) |
| HER2-targeted therapy | Yes | ＜0.001 | 1 | ＜0.001 | 1 | ＜0.001 | 1 |
|  | No | ＜0.001 | 5.117(2.532-10.342) | ＜0.001 | 2.666(1.687-4.215) | ＜0.001 | 2.791(1.714-4.545) |
|  | Unknown | ＜0.001 | 2.868(1.988-4.137) | ＜0.001 | 1.878(1.489-2.368) | ＜0.001 | 1.959(1.526-2.514) |
| Taxane type | Nab-paclitaxel | — | 1 | — | 1 | — | 1 |
|  | Other taxanes | 0.378 | 1.148(0.844-1.563) | 0.018 | 1.259(1.04-1.524) | 0.125 | 1.174(0.956-1.441) |
| COVID-19 | Pre-pandemic | 0.76 | 1 | 0.088 | 1 | 0.071 | 1 |
|  | Pandemic | 0.556 | 1.137(0.741-1.747) | 0.395 | 0.897(0.699-1.152) | 0.294 | 0.863(0.655-1.136) |
|  | Post-pandemic periods | 0.568 | 1.291(0.537-3.102) | 0.03 | 0.556(0.328-0.944) | 0.027 | 0.491(0.261-0.92) |

Supplementary Table S5. Complete Model 3 Cox regression results (all covariates).

| Covariate | SMD (Unmatched) | SMD (Matched) |
| --- | --- | --- |
| Year of diagnosis | -2.091 | -0.036 |
| COVID-19 | -1.658 | -0.006 |
| Age | 0.013 | -0.032 |
| Menstrual status | -0.026 | -0.006 |
| Clinical diagnosis | -0.023 | 0.025 |
| cT | -0.051 | 0.041 |
| cN | 0.019 | 0.049 |
| WHO grade | 0.274 | 0.056 |
| Hormone receptor (ER/PR) | 0.032 | 0.008 |
| HER-2 | 0.180 | 0.023 |
| Ki-67 | 0.162 | -0.002 |
| Breast surgery type | -0.130 | 0.014 |
| Axillary surgery type | 0.417 | 0.061 |
| Neoadjuvant chemotherapy | 0.321 | -0.079 |
| Endocrine therapy | 0.193 | -0.046 |
| HER2-targeted therapy | 0.461 | 0.007 |
| Radiotherapy | -0.235 | -0.040 |

Supplementary Table S6. Covariate balance (SMD) before and after PSM.

| Covariate | SMD (Unmatched) | SMD (Matched) |
| --- | --- | --- |
| Age | 0.197 | -0.045 |
| Menstrual status | 0.113 | -0.039 |
| Clinical diagnosis | -0.072 | 0.045 |
| cT | -0.077 | 0.051 |
| cN | -0.084 | 0.000 |
| WHO grade | -0.178 | 0.008 |
| Hormone receptor (ER/PR) | -0.125 | 0.016 |
| HER-2 | 0.038 | -0.027 |
| Ki-67 | -0.114 | 0.037 |
| Breast surgery type | -0.042 | -0.020 |
| Axillary surgery type | -0.136 | 0.048 |
| Neoadjuvant chemotherapy | 0.263 | 0.000 |
| Endocrine therapy | 0.256 | -0.057 |
| HER2-targeted therapy | 0.045 | -0.006 |
| Radiotherapy | 0.112 | -0.023 |

Supplementary Table S7. Covariate balance (SMD) before and after PSM in the overlap cohort (2018–2019).

| Subgroup |  | Total | Events | HR (95.0% CI) | P for Interaction |
| --- | --- | --- | --- | --- | --- |
| Age | ≤35 | 1050 | 164 | 1.25(0.67-2.333) | 0.830 |
|  | 36-45 | 2677 | 306 | 1.01(0.632-1.614) |  |
|  | 46-55 | 3312 | 352 | 1.103(0.724-1.679) |  |
|  | 56-69 | 2056 | 241 | 0.893(0.563-1.418) |  |
|  | ≥70 | 122 | 15 | — |  |
| cT | 0 | 27 | 2 | — | 0.569 |
|  | 1 | 2877 | 215 | 0.869(0.513-1.472) |  |
|  | 2 | 5063 | 612 | 0.976(0.728-1.309) |  |
|  | 3 | 719 | 106 | 1.052(0.49-2.26) |  |
|  | 4 | 309 | 83 | 1.22(0.575-2.589) |  |
|  | Unknown | 222 | 60 | 1.764(0.398-7.82) |  |
| cN | 0 | 4342 | 320 | 0.897(0.582-1.383) | 0.021 |
|  | 1 | 4288 | 580 | 1.067(0.775-1.468) |  |
|  | 2 | 236 | 74 | 5.498(0.656-46.061) |  |
|  | 3 | 323 | 87 | 0.836(0.419-1.666) |  |
|  | Unknown | 28 | 17 | — |  |
| WHO grade | 1 | 438 | 20 | 0.298(0.047-1.896) | 0.536 |
|  | 2 | 4707 | 479 | 1.081(0.774-1.509) |  |
|  | 3 | 1814 | 259 | 0.843(0.548-1.297) |  |
|  | Unknown | 2258 | 320 | 1.327(0.783-2.249) |  |
| Ki-67 | ≤14% | 1305 | 99 | 0.588(0.311-1.111) | 0.346 |
|  | 15%-50% | 5138 | 575 | 1.055(0.768-1.447) |  |
|  | ＞50% | 1929 | 254 | 1.462(0.924-2.315) |  |
|  | Unknown | 845 | 150 | 0.768(0.367-1.607) |  |
| Immunohistochemical subtyping | Luminal A | 971 | 63 | 0.617(0.288-1.324) | 0.000 |
|  | Luminal B | 3021 | 337 | 1.049(0.679-1.621) |  |
|  | HER2+(HR-) | 1190 | 157 | 1.776(0.975-3.233) |  |
|  | HER2+(HR+) | 1635 | 154 | 1.628(0.837-3.17) |  |
|  | Triple-negative | 1466 | 241 | 0.924(0.558-1.53) |  |
|  | Unknown | 934 | 126 | 0.661(0.302-1.448) |  |
| Breast surgery type | Breast-conserving surgery | 2036 | 152 | 0.973(0.564-1.679) | 0.834 |
|  | Mastectomy | 6263 | 869 | 1.044(0.802-1.359) |  |
|  | Mastectomy with reconstruction | 918 | 57 | 2.039(0.747-5.568) |  |
| Axillary surgery type | Sentinel lymph node biopsy | 2624 | 102 | 1.829(0.804-4.159) | 0.009 |
|  | Axillary lymph node dissection | 6593 | 976 | 0.977(0.77-1.238) |  |
| Neoadjuvant chemotherapy | Yes | 4569 | 619 | 0.992(0.757-1.302) | 0.518 |
|  | No | 4648 | 459 | 1.108(0.728-1.686) |  |
| Endocrine therapy | Yes | 3751 | 357 | 1.457(0.922-2.303) | 0.001 |
|  | No | 2753 | 411 | 1.091(0.765-1.558) |  |
|  | Unknown | 2713 | 310 | 0.564(0.376-0.845) |  |
| HER2-targeted therapy | Yes | 2181 | 170 | 1.538(0.94-2.515) | 0.014 |
|  | No | 5763 | 692 | 0.919(0.686-1.232) |  |
|  | Unknown | 1273 | 216 | 1.188(0.623-2.268) |  |
| Radiotherapy | Yes | 4168 | 639 | 1.148(0.851-1.548) | 0.018 |
|  | Unknown | 5049 | 439 | 0.913(0.627-1.33) |  |

Supplementary Table S8. Subgroup analyses for DFS.

| Subgroup |  | Total | Events | HR (95.0% CI) | P for Interaction |
| --- | --- | --- | --- | --- | --- |
| Age | ≤35 | 1050 | 141 | 1.172(0.594-2.31) | 0.903 |
|  | 36-45 | 2677 | 259 | 0.834(0.506-1.373) |  |
|  | 46-55 | 3312 | 303 | 0.985(0.63-1.541) |  |
|  | 56-69 | 2056 | 206 | 0.841(0.51-1.385) |  |
|  | ≥70 | 122 | 12 | — |  |
| cT | 0 | 27 | 2 | — | 0.684 |
|  | 1 | 2877 | 176 | 0.834(0.47-1.481) |  |
|  | 2 | 5063 | 527 | 0.891(0.646-1.229) |  |
|  | 3 | 719 | 95 | 0.841(0.396-1.786) |  |
|  | 4 | 309 | 78 | 1.175(0.513-2.691) |  |
|  | Unknown | 222 | 43 | 0.652(0.147-2.899) |  |
| cN | 0 | 4342 | 241 | 0.661(0.414-1.055) | 0.006 |
|  | 1 | 4288 | 517 | 1.056(0.749-1.49) |  |
|  | 2 | 236 | 71 | 5.133(0.616-42.761) |  |
|  | 3 | 323 | 80 | 0.72(0.354-1.463) |  |
|  | Unknown | 28 | 12 | — |  |
| WHO grade | 1 | 438 | 16 | 0.191(0.031-1.177) | 0.736 |
|  | 2 | 4707 | 403 | 0.907(0.639-1.287) |  |
|  | 3 | 1814 | 223 | 0.863(0.547-1.361) |  |
|  | Unknown | 2258 | 279 | 1.174(0.671-2.056) |  |
| Ki-67 | ≤14% | 1305 | 82 | 0.576(0.297-1.118) | 0.660 |
|  | 15%-50% | 5138 | 492 | 0.891(0.64-1.242) |  |
|  | ＞50% | 1929 | 217 | 1.435(0.875-2.352) |  |
|  | Unknown | 845 | 130 | 0.826(0.354-1.927) |  |
| Immunohistochemical subtyping | Luminal A | 971 | 51 | 0.684(0.314-1.489) | 0.001 |
|  | Luminal B | 3021 | 291 | 0.969(0.609-1.54) |  |
|  | HER2+(HR-) | 1190 | 132 | 1.642(0.84-3.208) |  |
|  | HER2+(HR+) | 1635 | 135 | 1.433(0.711-2.887) |  |
|  | Triple-negative | 1466 | 199 | 0.776(0.463-1.3) |  |
|  | Unknown | 934 | 113 | 0.682(0.294-1.581) |  |
| Breast surgery type | Breast-conserving surgery | 2036 | 113 | 0.802(0.433-1.483) | 0.911 |
|  | Mastectomy | 6263 | 771 | 1(0.759-1.319) |  |
|  | Mastectomy with reconstruction | 918 | 37 | 1.014(0.308-3.334) |  |
| Axillary surgery type | Sentinel lymph node biopsy | 2624 | 59 | 1.153(0.407-3.264) | 0.008 |
|  | Axillary lymph node dissection | 6593 | 862 | 0.936(0.726-1.206) |  |
| Neoadjuvant chemotherapy | Yes | 4569 | 548 | 0.972(0.726-1.3) | 0.934 |
|  | No | 4648 | 373 | 0.87(0.56-1.352) |  |
| Endocrine therapy | Yes | 3751 | 301 | 1.411(0.845-2.358) | 0.000 |
|  | No | 2753 | 342 | 0.976(0.669-1.424) |  |
|  | Unknown | 2713 | 278 | 0.513(0.333-0.792) |  |
| HER2-targeted therapy | Yes | 2181 | 141 | 1.408(0.817-2.426) | 0.010 |
|  | No | 5763 | 587 | 0.828(0.61-1.124) |  |
|  | Unknown | 1273 | 193 | 1.164(0.582-2.328) |  |
| Radiotherapy | Yes | 4168 | 564 | 0.995(0.731-1.353) | 0.069 |
|  | Unknown | 5049 | 357 | 0.871(0.576-1.315) |  |

Supplementary Table S9. Subgroup analyses for DMFS.

| Subgroup |  | Total | Events | HR (95.0% CI) | P for Interaction |
| --- | --- | --- | --- | --- | --- |
| Age | ≤35 | 1050 | 65 | 2.1(0.603-7.312) | 0.280 |
|  | 36-45 | 2677 | 119 | 0.73(0.38-1.404) |  |
|  | 46-55 | 3312 | 124 | 1.005(0.497-2.034) |  |
|  | 56-69 | 2056 | 121 | 0.78(0.389-1.565) |  |
|  | ≥70 | 122 | 10 | — |  |
| cT | 0 | 27 | 0 | — | 0.890 |
|  | 1 | 2877 | 77 | 0.737(0.349-1.555) |  |
|  | 2 | 5063 | 252 | 1.021(0.613-1.702) |  |
|  | 3 | 719 | 54 | 1.704(0.449-6.472) |  |
|  | 4 | 309 | 46 | 1.428(0.434-4.698) |  |
|  | Unknown | 222 | 10 | 0.378(0.046-3.107) |  |
| cN | 0 | 4342 | 107 | 0.876(0.445-1.726) | 0.151 |
|  | 1 | 4288 | 251 | 0.894(0.541-1.479) |  |
|  | 2 | 236 | 41 | — |  |
|  | 3 | 323 | 37 | 0.518(0.123-2.184) |  |
|  | Unknown | 28 | 3 | — |  |
| WHO grade | 1 | 438 | 4 | — | 0.000 |
|  | 2 | 4707 | 168 | 0.801(0.456-1.408) |  |
|  | 3 | 1814 | 126 | 0.791(0.414-1.511) |  |
|  | Unknown | 2258 | 141 | 1.717(0.792-3.722) |  |
| Ki-67 | ≤14% | 1305 | 41 | 0.567(0.233-1.38) | 0.233 |
|  | 15%-50% | 5138 | 219 | 1.18(0.695-2.002) |  |
|  | ＞50% | 1929 | 111 | 0.926(0.447-1.918) |  |
|  | Unknown | 845 | 68 | 1.71(0.433-6.762) |  |
| Immunohistochemical subtyping | Luminal A | 971 | 27 | 0.768(0.307-1.916) | 0.003 |
|  | Luminal B | 3021 | 118 | 1.289(0.558-2.976) |  |
|  | HER2+(HR-) | 1190 | 73 | 1.875(0.663-5.304) |  |
|  | HER2+(HR+) | 1635 | 54 | 2.157(0.741-6.282) |  |
|  | Triple-negative | 1466 | 111 | 0.551(0.277-1.097) |  |
|  | Unknown | 934 | 56 | 1.534(0.588-3.999) |  |
| Breast surgery type | Breast-conserving surgery | 2036 | 41 | 0.693(0.226-2.121) | 0.976 |
|  | Mastectomy | 6263 | 385 | 1.061(0.715-1.575) |  |
|  | Mastectomy with reconstruction | 918 | 13 | 0.789(0.055-11.335) |  |
| Axillary surgery type | Sentinel lymph node biopsy | 2624 | 25 | 0.544(0.152-1.944) | 0.727 |
|  | Axillary lymph node dissection | 6593 | 414 | 1.075(0.73-1.584) |  |
| Neoadjuvant chemotherapy | Yes | 4569 | 266 | 1.176(0.748-1.848) | 0.120 |
|  | No | 4648 | 173 | 0.717(0.391-1.317) |  |
| Endocrine therapy | Yes | 3751 | 98 | 2.672(1.004-7.114) | 0.002 |
|  | No | 2753 | 192 | 0.829(0.485-1.415) |  |
|  | Unknown | 2713 | 149 | 0.755(0.403-1.415) |  |
| HER2-targeted therapy | Yes | 2181 | 54 | 1.866(0.79-4.409) | 0.148 |
|  | No | 5763 | 280 | 0.757(0.485-1.181) |  |
|  | Unknown | 1273 | 105 | 2.048(0.779-5.388) |  |
| Radiotherapy | Yes | 4168 | 265 | 0.957(0.617-1.483) | 0.011 |
|  | Unknown | 5049 | 174 | 1.086(0.594-1.987) |  |

Supplementary Table S10. Subgroup analyses for OS.
